# Spontaneous perforation of jejunal gastrointestinal stromal tumor: A case report

**DOI:** 10.1016/j.ijscr.2020.06.088

**Published:** 2020-06-27

**Authors:** Ghassan T. Al-Swaiti, Mohammad H. Al-Qudah, Mohammad A. Al-Doud, Alaa R. Al-Bdour, Walid Al-Nizami

**Affiliations:** a Department of General Surgery, Jordanian Royal Medical Services (JRMS), Amman, Jordan; b Department of Radiology, Jordanian Royal Medical Services (JRMS), Amman, Jordan

**Keywords:** Acute abdomen, Perforation, Gastrointestinal stromal tumor

## Abstract

**Introduction:**

The incidence of small bowel tumors is comparatively rare when compared to colonic tumors. They comprise less than 10% of all gastrointestinal neoplasms. Gastrointestinal stromal tumor (GIST) is the most common mesenchymal neoplasm of the gastrointestinal tract; however it accounts for less than 1% of all gastrointestinal tumors.

**Aim:**

To report a rare case of jejunal gastrointestinal stromal tumor presenting as a life-threatening emergency in a young adult male who was treated in the General Surgery department in the Jordanian Royal Medical Services (JRMS), Amman, Jordan. PRESENTED CASE: We report a 59-year-old male who presented to our emergency department with severe generalized abdominal pain. The patient was in a hypovolemic shock. Abdominal examination revealed a massively distended, tender abdomen. Abdomenal CT scan with IV contrast showed significant fat stranding around the jejunum associated with pneumoperitoneum and free fluid. Exploratory laparotomy was emergently performed, revealing diffuse peritonitis secondary to perforated small bowel tumor. En bloc resection was performed. Histopathologic examination confirmed the presence of malignant gastrointestinal stromal tumor of the jejunum with R0 clearance.

**Discussion:**

Gastrointestinal stromal tumors are relatively rare and the jejunum is the least affected among other parts of the GI tract. The majority of ruptured GISTs occur spontaneously, and are located in the stomach and small bowel. Most ruptured GISTs are associated with dismal prognosis.

**Conclusion:**

A jejunal gastrointestinal stromal tumor can rarely present with spontaneous perforation. However, it is important to highlight this condition, as early emergency intervention constitutes the key to a good outcome.

## Highlights

• GIST is rare and its presentation as spontaneous rupture is extremely rare.
• GIST preoperative diagnosis remains difficult.
• Local excision of the GIST associated with the use of imatinib is effective.
• GIST is a rare pathological condition.

## 1 Introduction

Gastrointestinal Stromal Tumor (GIST) is the most common mesenchymal neoplasm of the gastrointestinal tract; however it accounts for less than 1% of all gastrointestinal tumors. It originates from the interstitial cells of Cajal, which are part of the autonomic nervous system of the intestine. The majority of the lesions are benign with a possibility of 20–30% for malignancy. It occurs mainly in the submucosal connective tissue of the stomach, small intestine, esophagus, colon, rectum, omentum and mesentery. It occurs more commonly in men with a median age of 50–70. GISTs are thought to be the result of mutations of proto-oncogene which encodes the cell surface tyrosine kinase receptor. Presenting symptoms in GISTs are non-specific and they are most often diagnosed incidentally [1]. This work is submitted in line with the SCARE criteria [10].

## 2 Case report

**Fig. 1:**
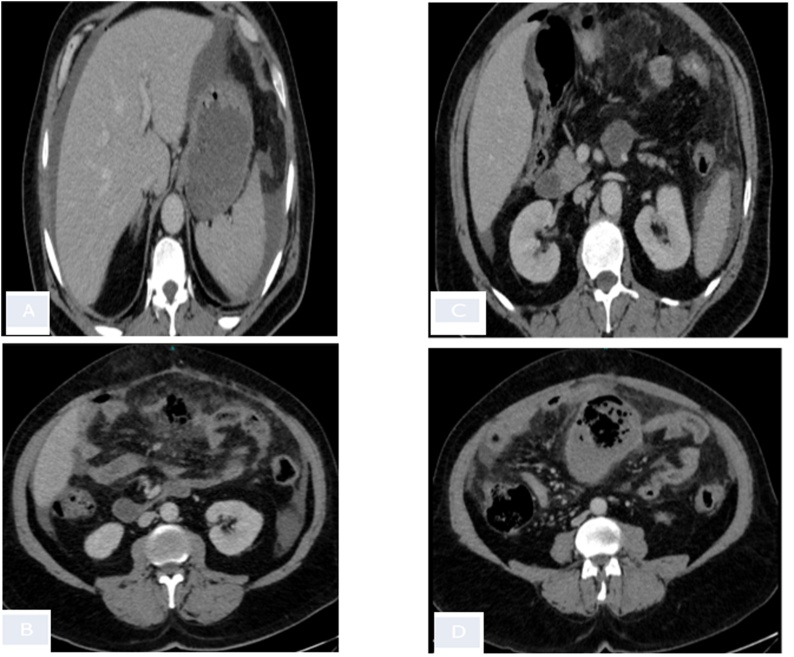
Abdominal CT scan. (A) Axial CT image demonstrated an intra-abdominal free fluid. (B) Axial CT image demonstrated a pneumoperitoneum and significant fat stranding in the small bowel mesentery. (C) Axial CT image demonstrated a pneumoperitoneum and significant fat stranding surrounding a jujenal segment of small bowel, which shoes gas locules at its wall representing the site of perforation. (D) Axial CT image demonstrated a focal hypo-attenuating wall thickening of small bowel segment and air fluid level.

A 59 years old male patient presented to our emergency department with severe generalized abdominal pain of four-day duration. The pain developed gradually, until it became more severe and unbearable. The patient had normal bowel habits. However, he had 3-month history of weight loss. On presentation, he appeared ill and shocked. His vital signs were: temperature 37.7 °C orally, heart rate 120/minute, respiratory rate 22/minute, and blood pressure 90/60 mm Hg. Abdominal examination revealed massively distended, tender rigid abdomen. Digital rectal examination revealed the presence of soft stool and it was negative for fresh blood or melena. His initial laboratory showed a hemoglobin of 11.1 g/dL, leukocyte count (10.3 × 10^9^)/L (Segmented neutrophils 70%), sodium (135 mEq/L) and potassium (3.5 mEq/L) with normal creatinine and blood urea nitrogen level (1 mg/dL, 29 mg/dL). An abdominal CT scan with IV contrast enhancement was performed after initial resuscitating, it showed pneumoperitoneum with significant fat stranding in the small bowel mesentery (see Fig. 1).

We decided to perform exploratory laparotomy. General anesthesia with endotracheal intubation was performed. Prophylactic antibiotics were administered. Nasogastric drainage and urinary catheter tubes had already been inserted.

**Fig. 2:**
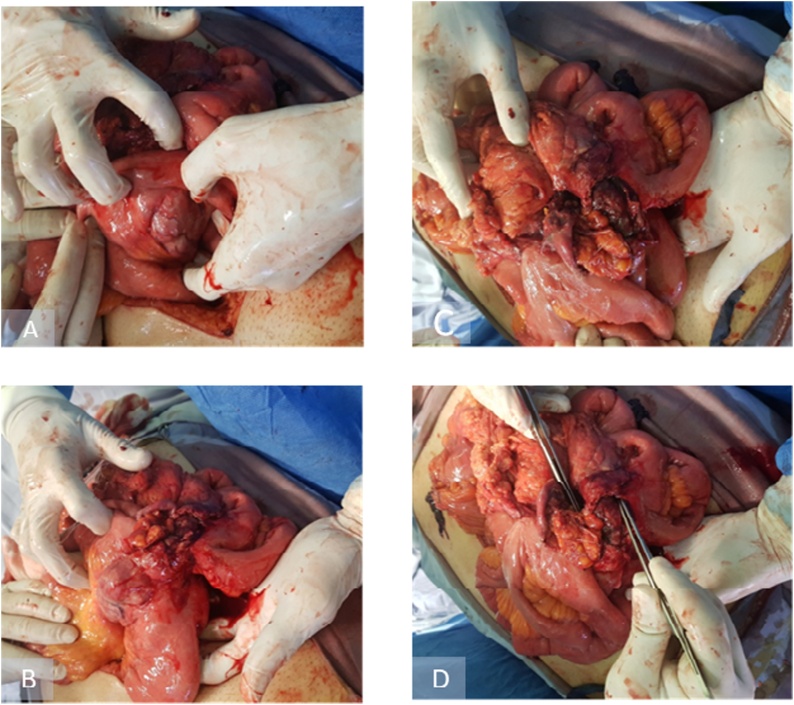
Intra operative photos demonstrating. (A) Hard mass arising from mid jejunum with extensive adhesion to other small bowel loops. (B) Mass of 10 cm arising from mid jejunum. (C) Extensive adhesion to other small bowel loops. (D) Site of perforation at the mesenteric side.

Upon entrance to the peritoneal cavity, a gush of turbid, free intraperitoneal fluid, mixed with enteric content came out. Suction of about 3000 mL was done. Exploration of the abdomen revealed the presence of a large mass lesion, measuring about 11 × 9 cm, arising from the mid jejunum with extensive adhesions involving the majority of small bowel length. This tumor was grossly perforated resulting in fecal peritonitis. The liver was grossly free of metastasis. No synchronous lesions were identified in the small or large bowel. Other intraabdominal organs were assessed and were grossly intact. Apart from the proximal 40 cm of the jejunum and the distal 30 cm of the ileum, the whole length of the small bowel was diseased and could not be preserved (See Fig. 2).

Keeping in mind the possibility of short bowel syndrome and its catastrophic consequences, creation of stoma at 40 cm from the DJ junction was not an option. Primary anastomosis was performed as the sole option of treatment at the expense of high possibility of anastomotic leak. Resection of the tumor along with the surrounding adhesive, unhealthy small bowel was done. The peritoneal cavity was generously irrigated with warm saline followed by aqueous betadine and finally warm saline again. A primary jejuno-ileal bowel anastomosis was performed using gastrointestinal staplers. Two intraperitoneal drains were applied and the abdomen was finally closed. The patient was extubated and transferred to the intensive care unit.

On day one post-surgery, the patient developed melena and coffee-ground gastric drainage on NG tube along with a drop in hematocrit level. His gastrointestinal bleeding responded to fluid resuscitation and blood products transfusion. On the fourth postoperative day, he started feeding and was transferred to the surgical ward. On the eleventh postoperative day, the patient was discharged home in an excellent general condition. On the 2-week post-surgery follow up, the patient had wound infection that required hospitalization for IV antibiotics and regular dressings.

Histopathologic examination of the specimen showed a malignant, spindle cell- type GIST with a mitotic rate of 8 MF/50 HPF. Histologic grade was high (G2). Adequate safety margins were confirmed. The number of lymph nodes retrieved in the specimen was 9. None of which was involved by the tumor. The pathologic stage was pT4 pN0.

One-month and two-month post-surgery follow up visits to the clinic were unremarkable. The patient maintained the same body weight as when discharged. No features of short bowel syndrome were detected. The patient started oral imatinib treatment 3 weeks post-surgery.

## 3 Discussion

GISTs are considered the most common mesenchymal tumors in the gastrointestinal tract [2] that occurs in adults more than 40 years of age, where they peak between 60 and 65 years. Males are affected more than females but without any geographic or ethnic relation [3,4]. The stomach is the most affected site followed by the small intestine, were 10% of the cases occur in the jejunum [3,5,6].

One third of the patients with GIST are asymptomatic [8] but it's not usual for patients with GIST of the jejunum to complain from abdominal pain, early satiety and abdominal fullness or to have a palpable mass [5,6].

Most of GIST are diagnosed incidentally during surgery, CT or endoscopy but several examinations of the gastrointestinal track failed to reach the correct diagnosis with 100% certainty [3,7]. To diagnose GISTs we depend on the morphology of the tumor cells and on immunohistochemistry. The morphological features are divided to spindle cell type 70%, epithelioid cell 20% and a mixed type 10%. Nearly all of GIST are positive for KIT (CD117) where 70% are positive for CD34 by immunohistochemistry [2]. Perforation of GIST is rare but if it happens the jejunum is the most commonly affected, causing hemoperitonium or abscess formation [5,6,8]. Surgical resection is the treatment of choice for GISTs, but there is not enough evidence to indicate an optimal resection margin size. However, a negative margin is important to prevent local recurrence. Lymph nodes involvement is rare, their dissection is not typically indicated. GISTs exceeding 10 mm adjuvant imatinib is recommended and it is the only effective drug for GISTs.

Imatinib gives a 14% absolute reduction in recurrence rate, achieving 97% recurrence-free survival [[2], [3], [4], [5], [6], [7], [8], [9]]. Our discussion continues whether to establish stomas or to preform intestinal anastomosis in the presence of peritonitis in an emergency settings. We bear in mind anastomotic dehiscence and metabolic derangements of small bowel proximal stomas. However, anastomosis is contraindicated in conditions where there is a high risk of leak such as fecal contamination or peritonitis or disseminated malignancy or unhealthy bowel conditions Despite the abovementioned, the decision of anastomosis due to the proximity of the jejunostomy was taken to avoid metabolic derangements [8]. Another area of debate is the use of peritoneal lavage and the different solutions that are used during the procedure. It is widely practiced, yet there is little evidence to show its benefits. What type of fluid, how much fluid should be used and what is the benefit achieved are all questions that need to be answered. Lavage with fluids may have a dilutional effect. Saline and water, may act as a physical cleaner, antibacterial agent or antiseptics, such as chlorhexidine and povidone iodine or antibiotics, may be toxic to bacteria or tumor cells by bacterial cell lysis [2,4,[6], [7], [8],^10^].

## 4 Conclusion

In conclusion, GIST is rare and its presentation as spontaneous rupture is extremely rare. Despite all the advancement in diagnostic procedures the preoperative diagnosis remains difficult. However, local excision of the tumor associated with the use of imatinib was effective. This case is remarkable for several reasons: (1) the combination of difficult decision to undertake in emergency settings. (2) The scarcity of this pathological condition. (3) The successful treatment without complications of this patient.

## Declaration of Competing Interest

None of the authors have conflicts of interest.

## Funding

This study has no sponsors and is self-funded.

## Ethical approval

Ethical approval has been taken from the ethical committe at King Hussein Medical Center, Amman, Jordan. The reference number is 19/12-2019.

## Consent

Written informed consent was obtained from the patient for publication of this case report and accompanying images. A copy of the written consent is available for review by the Editor-in-Chief of this journal on request.

## Author contribution

**Category 1**: Pre-Operative care: Emergency department care: Ghassan T. Alswaiti.

Interpretation of the CT images: Walid Al-Nizami.

Operation: Main Surgeon: Ghassan T. Alswaiti.

Assisstant Surgeon: Mohammad A. Al-Doud.

Post-Operative Care: Ghassan T. Al-Swaiti, Mohammad H. Al-Qudah, Mohammad A. Al-Doud and Alaa R. Al-Bdour.

**Category 2**: Drafting the manuscript: Ghassan T. Al-Swaiti, Mohammad H. Al-Qudah revising the manuscript critically for important intellectual content: Mohammad A. Al-Doud, Alaa R. Al-Bdour and Walid Al-Nizami.

**Category 3**: Approval of the version of the manuscript to be published (the names of all authors must be listed): Ghassan T. Al-Swaiti, Mohammad H. Al-Qudah, Mohammad A. Al-Doud, Alaa R. Al-Bdour and Walid Al-Nizami.

## Registration of research studies

Does not need registration.

## Guarantor

Dr. Ghassan T. Al-Swaiti.

## Provenance and peer review

Not commissioned, externally peer reviewed.

## References

[1] Cuschieri A., Hanna G. (2015). Essential Surgical Practice: Higher Surgical Training in General Surgery.

[2] Nishida T., Blay J.Y., Hirota S., Kitagawa Y., Kang Y.K. (2016). The standard diagnosis, treatment, and follow-up of gastrointestinal stromal tumors based on guidelines. Gastric Cancer.

[3] Sankey R.E., Maatouk M., Mahmood A., Raja M. (2015). Case report: jejunal gastrointestinal stromal tumour, a rare tumour, with a challenging diagnosis and a successful treatment. J. Surg. Case Rep..

[4] Roy S.D., Khan D., De Krishna K., De U. (2012). Spontaneous perforation of jejunal gastrintestinal stromal tumour (gist). Case report and review of literature. World J. Emerg. Surg..

[5] Misawa S.I., Takeda M., Sakamoto H., Kirii Y., Ota H., Takagi H. (2014). Spontaneous rupture of a giant gastrointestinal stromal tumor of the jejunum: a case report and literature review. World J. Surg. Oncol..

[6] Sato K., Tazawa H., Fujisaki S., Fukuhara S., Imaoka K., Hirata Y., Takahashi M., Fukuda S., Kuga Y., Nishida T., Sakimoto H. (2017). Acute diffuse peritonitis due to spontaneous rupture of a primary gastrointestinal stromal tumor of the jejunum: a case report. Int. J. Surg. Case Rep..

[7] Rana I., Sorokhaibam J. (2016). Spontaneous perforation of jejunal gastrointestinal stromal tumour presenting with multiple intraperitoneal abscess cavities: a case report and review of literature. Int. J. Sci. Rep..

[8] Alessiani M., Gianola M., Rossi S., Perfetti V., Serra P., Zelaschi D., Magnani E., Cobianchi L. (2015). Peritonitis secondary to spontaneous perforation of a primary gastrointestinal stromal tumour of the small intestine: a case report and a literature review. Int. J. Surg. Case Rep..

[9] Rasslan S., Fonoff A.M., Soldá S.C., Casaroli A.A. (1995). Ostomy or intestinal anastomosis in cases of peritonitis. Sao Paulo Med. J..

[10] Agha R.A., Borrelli M.R., Farwana R., Koshy K., Fowler A., Orgill D.P., For the SCARE Group (2018). The SCARE 2018 statement: updating consensus Surgical CAse REport (SCARE) guidelines. Int. J. Surg..

